# Enhancement of 3-MA in Paclitaxel Treatment of MDA-MB-231 Tumor-Bearing Nude Mice and Its Mechanisms

**DOI:** 10.3390/ijms26136191

**Published:** 2025-06-27

**Authors:** Jing Wang, Zhe Xiong, Yaowen Liu, Muhammad Ameen Jamal, Xia Wang, Chang Yang, Ziyi Gu, Xiaojing Chen, Jingjing Xiong, Yubo Qing, Honghui Li, Kaixiang Xu, Hong-Jiang Wei, Hong-Ye Zhao

**Affiliations:** 1 Yunnan Province Key Laboratory for Porcine Gene Editing and Xenotransplantation, Yunnan Agricultural University, Kunming 650201, China; kmwangjing@163.com (J.W.); xiongzhe4127@163.com (Z.X.); yaowenliu@foxmail.com (Y.L.); drameen007@gmail.com (M.A.J.); wangxia20211221@163.com (X.W.); theurgytheurgy@163.com (C.Y.); 13187668896@163.com (Z.G.); 2023110002@stu.ynau.edu.cn (X.C.); 2022210444@stu.ynau.edu.cn (J.X.); qingyubo20@163.com (Y.Q.); honghui8300@aliyun.com (H.L.); tsljmuch@163.com (K.X.); 2 Yunnan Province Xenotransplantation Research Engineering Centre, Yunnan Agricultural University, Kunming 650201, China; 3 Faculty of Animal Science and Technology, Yunnan Agricultural University, Kunming 650201, China; 4 College of Veterinary Medicine, Yunnan Agricultural University, Kunming 650201, China; 5 Yunnan Laboratory of Molecular Biology of Domestic Animals, Kunming Institute of Zoology, Chinese Academy of Sciences, Kunming 650223, China; 6 College of Food Science and Technology, Yunnan Agricultural University, Kunming 650201, China

**Keywords:** MDA-MB-231, triple-negative breast cancer, paclitaxel, autophagy, 3-MA

## Abstract

Triple-negative breast cancer (TNBC) poses significant challenges due to its high aggressiveness, poor prognosis, and the lack of effective targeted therapies. Paclitaxel (PTX) is a chemotherapeutic agent commonly used in the treatment of TNBC; however, its efficacy is often compromised by drug resistance mediated by autophagy. This study investigated the synergistic effects of the autophagy inhibitor 3-methyladenine (3-MA) and PTX in a TNBC nude mouse model. Monitoring tumor volume and employing HE staining, immunofluorescence, and transmission electron microscopy revealed that PTX monotherapy induced tumor autophagy, characterized by the accumulation of LC3B/VPS34 proteins and an increase in autophagosomes. However, the co-administration of 3-MA reversed this process, significantly decreasing the tumor growth rate. Immunofluorescence and qPCR demonstrated that the combination group had fewer Ki-67-positive cells and more Caspase-3-positive cells, along with upregulated expression of autophagy-related genes and Caspase-family apoptosis genes. Consequently, this study suggests that inhibiting autophagy with 3-MA disrupts the autophagy-mediated protective mechanism of tumor cells, promoting the activation of apoptotic signals and enhancing the antitumor activity of PTX. These findings may offer new molecular mechanistic insights and potential therapeutic strategies for overcoming PTX resistance in TNBC.

## 1. Introduction

Triple-negative breast cancer (TNBC) is recognized as the most aggressive and hazardous form of breast cancer. Due to the absence of the following three primary therapeutic targets: estrogen receptors, progesterone receptors, and HER-2, the TNBC is characterized by significant invasiveness, recurrence and metastasis, and a poor prognosis [1]. It accounts for approximately 15% of all breast cancer cases [2]. Notably, less than 10% of patients experience tumor reduction following conventional treatment, primarily due to lack of progression after multiple treatment cycles [3]. Currently, treatment options for non-metastatic diseases are confined to chemotherapy, underscoring the urgent need for novel therapeutic strategies [4,5,6]. Paclitaxel (PTX) is a natural anticancer drug extracted from plants of the genus Taxus. It inhibits tumor cell mitosis by disrupting the physiological function of cellular microtubules, ultimately leading to tumor cell death. PTX is a chemotherapy drug widely used for the treatment of TNBC, but its efficacy was limited by drug resistance. During chemotherapy treatment, the development of tumor drug resistance poses an inevitable challenge. Rather than simply increasing the dosage of chemotherapy drugs or expanding the range of chemotherapeutic agents to suppress cancer, it is more imperative to investigate the mechanisms of cancer cell drug resistance and formulate rational targeted therapy strategies.

Autophagy promotes tumor progression by facilitating cellular adaptation to various environmental stresses, including hypoxia, nutrient deprivation, and cancer therapy [7]. It plays a crucial role in maintaining redox balance and energy homeostasis, with its inhibition capable of preventing the transformation of precancerous lesions into invasive tumors—a mechanism partially dependent on sustaining mitochondrial metabolism [8]. Cancer cells induce and rely on autophagy for survival; just as normal cells sustain life through autophagy during starvation, cancer cells similarly exploit autophagy-mediated recycling mechanisms to preserve mitochondrial function and energy homeostasis to meet their heightened metabolic demands for growth and proliferation. Previous studies have confirmed the pivotal role of autophagy in cancer progression [9,10,11,12]. This suggests that modulating autophagy may serve as an effective intervention strategy for cancer treatment [13], highlighting the potential therapeutic advantage of autophagy inhibition in oncology [14]. Consequently, inhibiting autophagy may enhance the efficacy of paclitaxel in treating triple-negative breast cancer patients. Thus, targeting autophagy may represent a promising therapeutic strategy.

Commonly employed autophagy inhibitors include Bafilomycin A1 (Baf-A1), 3-methyladenine (3-MA), and wortmannin. 3-MA inhibits autophagy by suppressing the activity of PI3K. Specifically, 3-MA primarily inhibits the activity of class I and class III PI3K, with a particularly significant effect on class III PI3K. In the early stages of autophagy, class III PI3K forms complexes with proteins such as Beclin-1, which promotes the formation of phagosomes. When 3-MA enters the cell, it binds to specific domains of class III PI3K, altering its spatial conformation. This prevents class III PI3K from catalyzing the conversion of phosphatidylinositol to phosphatidylinositol-3-phosphate, thereby hindering the formation and expansion of phagosomes [15]. Studies have demonstrated that 3-MA, in combination with hypoxia, can suppress autophagy and promote apoptosis in HCT-116 colorectal cancer cell model. This suggests that autophagy may confer resistance to anti-tumor drugs and exert detrimental effects on tumors, thereby providing certain protective effects [16]. Zheng et al. reported in their study on human breast cancer cells MCF-7 that 3-MA effectively inhibits cellular autophagy and promotes apoptosis [17]. The findings suggest that hypoxia alone, in vitro, increases autophagy and apoptosis in tumor cells. In contrast, the combined treatment with 3-MA significantly suppresses hypoxia-induced autophagy and enhances hypoxia-induced apoptosis, highlighting the role of 3-MA as a classical autophagy inhibitor. It primarily hinders the formation and maturation of autophagosomes and is effective in inhibiting autophagy. Chen et al. employed 3-MA to suppress autophagy in hepatocellular carcinoma cells. The results suggest that suppressing autophagy hinders excessive lipid accumulation in free-fatty-acid-induced liver cancer cells and hepatocytes [18].

In this study, a TNBC nude mouse model was developed using MDA-MB-231 cells and tumor mass tissue transplantation to investigate the synergistic effects of the autophagy inhibitor 3-MA and PTX. The tumor volume and body weight were measured. Additionally, HE staining, immunofluorescence, transmission electron microscopy, and q-PCR tests were employed to detect and assess the occurrence of autophagy and its impact on tumor cell apoptosis following autophagy inhibition. This may provide a theoretical basis for improving PTX resistance in TNBC treatment.

## 2. Results

### 2.1. Establishment of TNBC Nude Mouse Model

**Figure 1:**
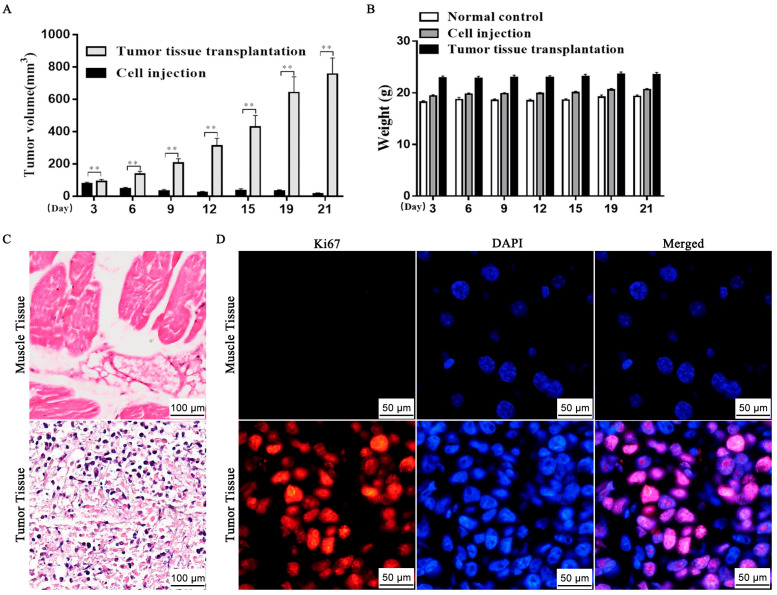
Comparison of tumor growth and body weight curve in MDA-MB-231 cell-transplanted and tumor-mass-transplanted nude mice. (**A**) Statistics on tumor volume in MDA-MB-231 cell-transplanted and mass-transplanted group. ** p < 0.01 indicates a significant difference; (**B**) Changes in body weight of normal control group, cell-transplanted group and mass-transplanted group; (**C**) HE staining images of adjacent muscle tissue and tumor tissues; (**D**) Immunofluorescence images of Ki-67 in muscle and tumor tissues.

The TNBC nude mouse model was first constructed through subcutaneous injection of MDA-MB-231 cells. A mass was observed on the third day after the subcutaneous injection of MDA-MB-231 cells. However, the success rate of tumor-bearing mouse models obtained through injection methods is relatively low. The tumor tissue from one of the mice was harvested and cut into small pieces, which were then transplanted subcutaneously into healthy nude mice. Thus, we obtained a batch of tumor-bearing mice with high tumor formation rate and consistency through tumor mass transplantation. The tumor volume in the tumor mass transplantation group increased significantly compared to the cell injection group (*p* < 0.01) (Figure 1A). However, the body weight of the mice in all three groups remained unchanged (Figure 1B). The histological examination of breast cancer tumor tissue and normal muscle tissue, as evidenced by HE staining (Figure 1C), revealed that normal muscle tissue displayed well-differentiated muscle cells, whereas the breast cancer mass tissue exhibited loose intercellular connections, poorly differentiated cells with enlarged nuclei, uneven chromatin staining, and vacuolated nuclei. Additionally, the number of Ki-67 positive cells in the tumor tissues was significantly higher than that of normal muscle tissue (Figure 1D). These findings indicate that the tumor tissue was highly heterogeneous and exhibited vigorous proliferation. Consequently, a nude mouse subcutaneous transplant tumor model was successfully established.

### 2.2. Effects of Combined Use of PTX and 3-MA on Tumor Treatment in Tumor-Bearing Nude Mice

**Figure 2:**
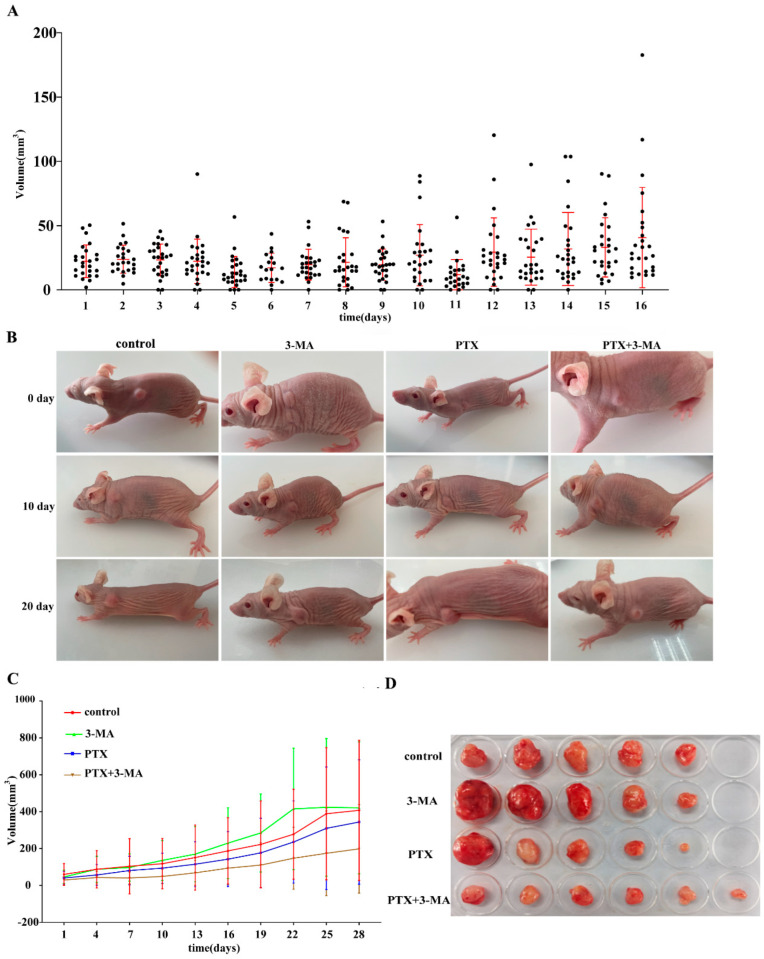
Effect of tumor volume on the therapeutic efficacy of paclitaxel in tumor−bearing nude mice. (**A**) Tumor volume measurements of mass-transplanted tumor models over 16 days; (**B**) Tumor changes on days 0, 10, and 20 during treatment in tumor-bearing nude mice. (**C**) Tumor volume statistics of nude mice treated with PTX, 3-MA, and PTX + 3-MA for 28 days (*n* = 10); (**D**) Subcutaneous tumor mass dissection in tumor-bearing nude mice.

To further assess the synergistic effects of PTX and 3-MA, the MDA-MB-231 breast cancer model was established in another cohort of nude mice via tumor tissue mass transplantation (Figure 2A). On the 16th day post-transplantation, PTX and 3-MA were administered via intraperitoneal injection, and the tumor growth curves for each group of nude mice were subsequently plotted (Figure 2B,C). At 28 days post-treatment, the subcutaneous tumor tissues from each group were dissected and images were collected for documentation (Figure 2D). Comparing the tumor growth curves and the volume of dissected tumor tissue masses among the groups, the results indicated that the tumor growth rate in the group receiving both PTX and 3-MA was slower than PTX alone group. These findings suggest that autophagy inhibition can decelerate tumor growth and enhance the effects of PTX in TNBC.

### 2.3. HE Staining of Tumor and Organ Tissues in MDA-MB-231 Tumor-Bearing Nude Mice After Combined Treatment of PTX and 3-MA

**Figure 3:**
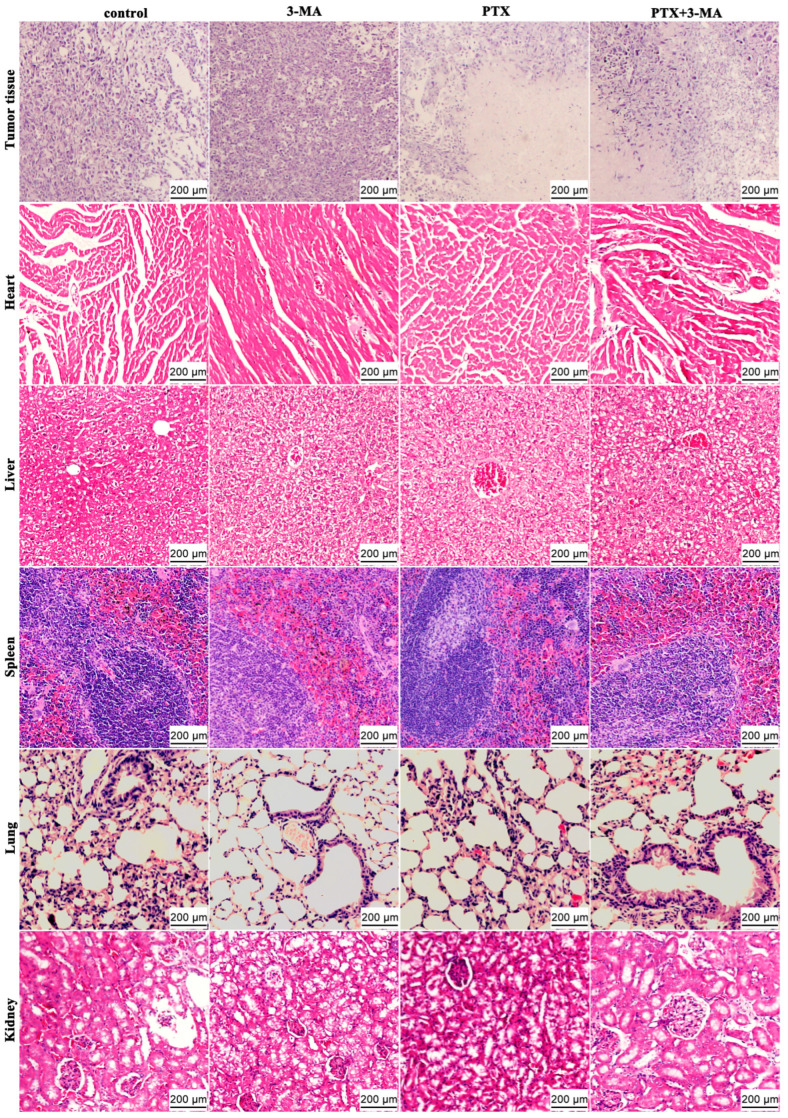
HE staining of tumor masses and organ tissues in MDA-MB-231 tumor-bearing nude mice treated with PTX and 3-MA. The morphology of the tumor, heart, liver, spleen, lung, and kidney tissues in the Control, PTX, 3-MA, and PTX + 3-MA groups were observed through HE staining. Scale bar = 200 μm.

The histological analysis of tumor mass and organs were observed by HE staining. The tumor tissue exhibited an increased nucleocytoplasmic ratio, tightly packed cells, and abnormal, irregular morphological structures. The nuclei predominantly displayed pathological mitotic phases without differentiation (Figure 3). Compared with the control group, there were no significant structural changes observed in the tumor tissue of the 3-MA group. However, necrotic tissue was observed in the tumor tissues of both the PTX group and the PTX + 3-MA group, with clearly demarcated necrotic foci. The necrotic areas exhibited cytoplasmic shrinkage forming vacuoles, relatively loose arrangement, and nuclear shrinkage into round or oval shapes.

The hepatic cells in each group were arranged along the central vein to form hepatic cords, with abundant glycogen accumulation observed in the cytoplasm. Extensive vacuolar degeneration was present in the cells, accompanied by blurred cell boundaries, presenting a mosaic-like appearance. The nuclei of the hepatocytes were reduced in size, and the hepatic sinusoids were widened with erythrocyte stasis. However, no inflammatory cell infiltration or any cancerous cells or tissues were identified. The cells in the tissues of the heart, spleen, lung, and kidney are arranged densely and orderly, with clear cellular structures and outlines. The characteristic structures of myocardium, splenic trabeculae, alveoli, bronchioles, and renal glomeruli are distinct, and the morphological structures are essentially normal. The walls of blood vessels and lymphatic vessels between tissues appear normal, with no abnormal cells or tissues observed in the interstitium. These results indicate that the tumor has not metastasized.

### 2.4. Effects of 3-MA on the PTX Induced Autophagy in MDA-MB-231 Tumor-Bearing Nude Mouse

**Figure 4:**
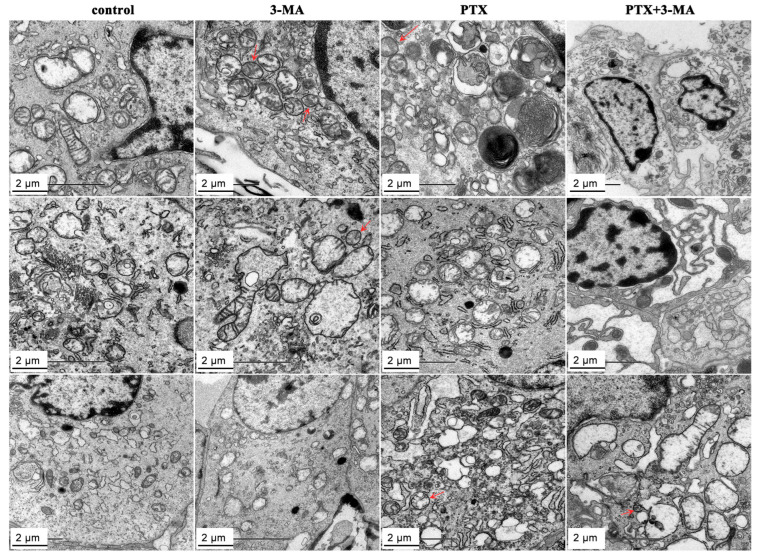
Transmission electron microscopy images of tumor tissues in MDA-MB-231 tumor-bearing nude mice treated with PTX and 3-MA. The tumor tissues autophagosomes in the control, PTX, 3-MA, and PTX + 3-MA groups were observed through transmission electron microscope. Scale bar = 2 μm. The structure indicated by the red arrow is an autophagosome.

**Figure 5:**
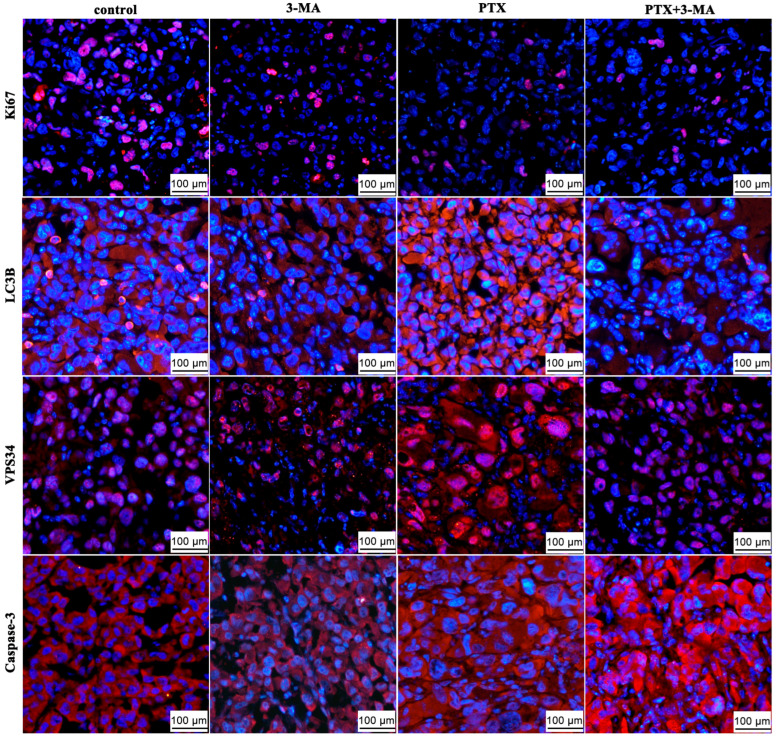
The effects of PTX and 3-MA combination on the expression of Ki-67, LC3B, VPS34, and Caspase-3 in tumor tissues. The expression of Ki-67, LC3B, VPS34, and Caspase-3 in the tumor tissues for the control, PTX, 3-MA, and PTX + 3-MA groups were detected by immunofluorescence. Scale bar = 100 μm.

To assess the impact of 3-MA on autophagy inhibition, the expression of associated proteins in tumor tissues following the administration of PTX and 3-MA was examined using transmission electron microscope (Figure 4) and immunofluorescence staining (Figure 5). Compared to the control group, the number of autophagic vesicles and LC3B, VPS34-positive cells increased in the tumor tissues of PTX group. Contrarily, the PTX, PTX + 3-MA groups exhibited a reduction in the number of autophagic vesicles and LC3B, VPS34-positive cells, and a significant increase in Caspase-3-positive cells in tumor tissues. Immunofluorescence analysis demonstrated that PTX treatment led to a reduction in the fluorescence intensity of Ki-67 in tumor tissue, while LC3B, VPS34, and caspese-3-positive cells accumulated in tumor tissue. However, the fluorescence intensity of the LC3B positive cells in the PTX + 3-MA group were reduced because of 3-MA treatment. These findings indicated that 3-MA effectively inhibited PTX-induced autophagy in tumor cells by promoting cell apoptosis. These results indicate that 3-MA promotes apoptosis by inhibiting PTX induced autophagy in tumor cells.

### 2.5. Gene Expression in MDA-MB-231 Tumor-Bearing Nude Mice After Combined Treatment of PTX with 3-MA

**Figure 6:**
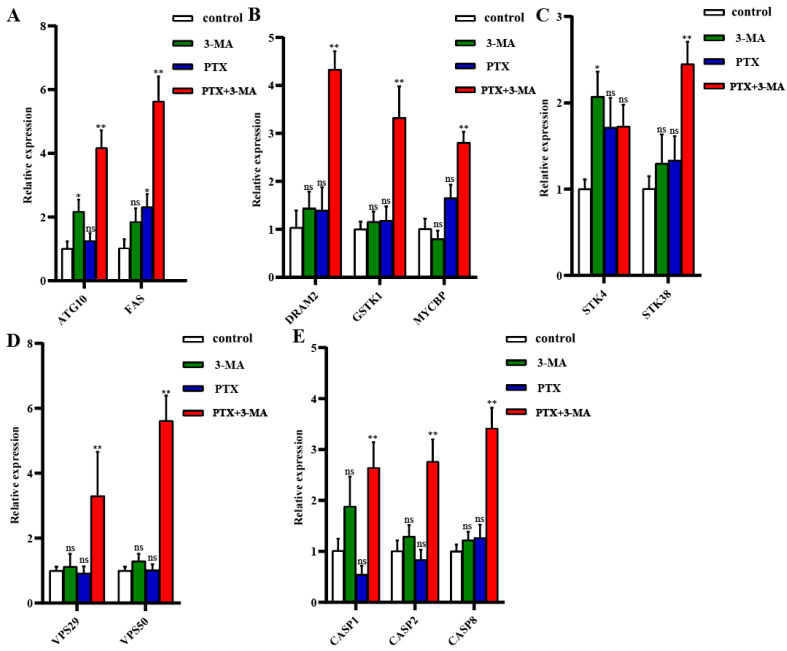
Expression of related genes in MDA-MB-231 tumor-bearing nude mice treated with PTX and 3-MA combination. (**A**) mRNA expression levels of *ATG10* and *FAS*; (**B**) mRNA expression levels of *DRAM2, GSTK1*, and *MYCBP*; (**C**) mRNA expression levels of *STK4* and *STK38*; (**D**) mRNA expression levels of *VPS29* and *VPS50*; (**E**) mRNA expression levels of *CASP1*, *CASP2*, and *CASP8*. *GAPDH* was used as the internal reference; *n* = 3, * *p* < 0.05 or ** *p* < 0.01 indicates a significant difference. ^ns^ *p* > 0.05 indicates no significant differences.

To investigate the molecular mechanisms underlying the combined treatment of PTX and the autophagy inhibitor 3-MA, the mRNA expression of genes associated with autophagy and apoptosis-related signaling pathways were quantified using RT-qPCR. In the context of the autophagy signaling pathway, quantitative analysis revealed an increase in the mRNA expression level of *ATG10* in the 3-MA group, and of the *FAS* gene in PTX treatment group (*p* < 0.05). While, the mRNA levels of both genes were significantly increased in PTX with autophagy inhibitors (PTX+3MA) (*p* < 0.01) (Figure 6A). The mRNA levels of *DRAM2*, *GSTK1*, and *MYCBP* genes remained unchanged only in the treatment groups of 3-MA and PTX treatment (*p* > 0.05), while significantly increased in combined treatment groups (*p* < 0.01; Figure 6B). Within the STK family, the mRNA level of the *STK38* gene was significantly elevated after the combined treatment of PTX with an autophagy inhibitor (*p* < 0.01; Figure 6C). In the VPS family, the mRNA expression levels of *VPS29* and *VPS50* genes did not exhibit significant changes when treated with autophagy inhibitors or PTX alone. However, their expression levels were significantly increased when PTX was used in combination with 3-MA (*p* < 0.01; Figure 6D). The caspase family plays a pivotal role in the induction, transduction, and amplification of intracellular apoptotic signaling. The mRNA levels of *CASP1*, *CASP2*, and *CASP8* genes did not show significant changes in the alone treatment of PTX or 3-MA (*p* > 0.05); however, their expression significantly increased in combined treatment of 3-MA and PTX (*p* < 0.01; Figure 6E).

## 3. Discussion

Paclitaxel (PTX), as a first-line chemotherapy drug, is relatively effective and safe for the treatment of breast cancer; however, its efficacy is limited by resistance [19]. Autophagy is one of the mechanisms underlying PTX resistance, but the key molecular mechanism of PTX-induced autophagy remains unclear. Moreover, therapeutic approaches to ameliorate PTX resistance still require further investigation. Thus, in this study, the synergistic effect of the autophagy inhibitor 3-MA on PTX and its mechanism were investigated in a TNBC nude mouse model, aiming to provide a theoretical basis for exploring new strategies to overcome PTX resistance. By observing the tumor growth, survival status, and related molecular changes in TNBC nude mice, we can comprehensively evaluate the impact of the combination of 3-MA and PTX. Moreover, through further in-depth analysis of the signal pathways and molecular mechanisms involved in this process, we may discover potential therapeutic targets. This not only helps to understand the role of autophagy in PTX resistance but also paves the way for the development of more effective treatment regimens for TNBC patients.

In the advanced stages of cancer, rapid proliferation of tumor cells requires abundant nutrients under hypoxic and metabolic conditions, especially for low-vascularized solid tumors. Autophagy is a highly conserved self-degradation process that plays a key role in cellular stress response and survival [20]. In various types of cancer, including TNBC, tumor cells rely on autophagy as a nutrient source during their growth process. Therefore, autophagy plays a significant role in tumor cell survival and recurrence after chemotherapy. After radiotherapy, damaged tumor cells can eliminate impaired proteins and organelles by activating autophagy to store energy and maintain cell survival [21]. PTX treatment can induce autophagy in TNBC cells, which is an important mechanism of PTX resistance [22]. LC3B is a protein marker of autophagy [23]. In this study, LC3B-positive cells (Figure 5) and the autophagosomes (Figure 4) increased in the PTX-treated group, indicating that PTX therapy activated autophagy in TNBC cells, which is consistent with our previous study [24]. Therefore, inhibiting autophagy is a potential way to increase sensitivity and eliminate resistance to restore efficacy. Ki-67 is one of the most reliable biological indicators for detecting tumor cell proliferative activity [25]. In the present study, we also find that the Ki-67-positive cells decreased in the tumor tissue of the PTX + 3-MA group (Figure 5). This indicates that the combination with 3-MA enhances the effects of PTX on TNBC cell proliferation. Furthermore, we have also noticed the increase in caspase-3-positive cells in the immunofluorescence test and the increase in *CASP1*, *CASP2*, and *CASP8* mRNA level in PTX+3-MA group (Figure 6E). This indicates that the combination of 3-MA and PTX promotes apoptosis in TNBC mice.

As a classic autophagy inhibitor, 3-MA primarily blocks the PI3K signaling pathway to inhibit autophagosome formation, thereby disrupting the protective mechanism of tumor cells, promoting the activation and execution of apoptotic genes and enhancing the antitumor effect of PTX. Similar mechanisms have been observed in other studies. For instance, the combination of AKT inhibitors with PTX in treating TNBC significantly enhances the efficacy of PTX by inhibiting the PI3K/AKT/mTOR pathway [26], indicating that targeting autophagy-related signaling pathways is an effective strategy to overcome PTX resistance. In this study, we also observed that the combination of 3-MA and PTX significantly reduced TNBC tumor volume compared to PTX treatment alone, with evident patchy necrosis and nuclear shrinkage in tumor tissues, suggesting a marked enhancement of apoptosis.

*VPS29* and *VPS50* belong to the VPS family and are involved in the fusion of autophagosomes with lysosomes [27]. Their expression changes reflect the dynamic regulation of autophagic flux, which is closely related to paclitaxel-induced cellular stress responses (Figure 6D). This suggests that the combination treatment affects the fusion process of autophagosomes and lysosomes, thereby disrupting the ability of tumor cells to maintain homeostasis through autophagy. *DRAM2*, another key factor in the fusion of autophagosomes and lysosomes [28], exhibited upregulated expression (Figure 6B), further confirming that autophagic flux is blocked during combination therapy, leading to the disruption of the tumor cell’s autophagy-mediated protective mechanism.

FAS gene is a key receptor in the extrinsic apoptotic pathway [29]. In this study, the mRNA level of the *FAS* gene increased significantly (Figure 6A), indicating that autophagy inhibition removes the protective mechanism of tumor cells and promotes the activation of apoptotic signals. Concurrently, the expression of key apoptotic genes, including *CASP1*, *CASP2*, and *CASP8* (Figure 6E), was significantly upregulated after 3-MA and PTX combination treatment, further confirming that autophagy inhibition disrupts the protective mechanism of tumor cells, promotes apoptotic signal activation, and enhances paclitaxel-induced apoptosis in breast cancer cells. Additionally, *STK4* and *STK38* are critical serine/threonine kinases regulating the cell cycle and apoptosis [30]; they also exhibited an upregulation trend in the PTX+3-MA group, suggesting that 3-MA may induce tumor cell cycle arrest and apoptosis, thereby enhancing drug efficacy. This aligns with the established role of STK family members in regulating tumor cell fate [31]. *GSTK1* and *MYCBP* were involved in intracellular redox regulation and MYC-mediated transcriptional activity, respectively. In the q-PCR test, we also find that the mRNA level of *GSTK1* (Figure 6C) and *MYCBP* (Figure 6B) increased significantly in the PTX+3-MA group. This indicated that in 3-MA combination therapy, the membrane transport, redox balance, and transcriptional regulation processes were reprogrammed to effectively block tumor cell proliferation [32,33].

Therefore, in this study, blocking autophagy with 3-MA disrupted the autophagy-mediated protective mechanism of tumor cells, promoting apoptotic signal activation and enhancing the antitumor activity of PTX. This effectively counteracts PTX resistance mechanisms and delays the development of drug resistance. These findings are highly consistent with current research trends targeting autophagy-related signaling pathways to overcome PTX resistance, providing new molecular mechanistic insights and potential therapeutic strategies for addressing chemotherapy resistance in TNBC.

## 4. Materials and Methods

### 4.1. Cell Culture

The frozen MDA-MB-231 breast cancer cell line (National Collection of Authenticated Cell Cultures, Shanghai) was thawed in water bath at 37 °C for 3 min, the DMEM culture medium was added and centrifuged at 1500 rpm for 3 min to wash out the frozen liquid, and the cells were incubated at 37 °C, 5% CO_2_ in a petri dish containing DMEM culture medium with 10% fetal bovine serum (FBS) and 1% Penicillin-streptomycin antibiotic. The culture medium was changed every other day, and after 1 week of culture, the cells were counted and prepared for inoculation.

### 4.2. Animals 

The nude mice (BALB/c, Female, 4–6 weeks, 15 g–20 g) used in this study were provided by the Experimental Animal Department of Kunming Medical University. The experiments were conducted in accordance with the 3Rs principles. This study was approved by the Animal Ethics Committee of Yunnan Agricultural University, project number YNAU202105009, approval date: 31 May 2021. 

### 4.3. Establishment of TNBC Mouse Model

Healthy nude mice were adaptively raised under constant conditions for one week. Then 20 BALB/c nude mice were randomly selected and divided into experimental groups and control groups. Firstly, the TNBC mouse model was established by MAD-MB-231 cell transplantation. Briefly, the experimental group was subcutaneously injected with 200 μL of MDA-MB-231 cell suspension (5 × 10^6^ cells) in the groin region, while the control group was injected with same volume of PBS at the corresponding location. When the tumor grew to approximately 1000 mm^3^, one of the tumor-bearing mice was selected and anesthetized using 3% Sodium pentobarbital. Then the tumor masses were peeled off and soaked in DMEM. Next the tumor was cut into approximately 1 mm^3^ mass. The healthy female nude mice were anaesthetized using 3% sodium pentobarbital, and the tumor mass was transplanted subcutaneously through inoculation trocar. The body weight and tumor volume of the nude mice in each group were measured every 2 days starting from 4 days post-inoculation. The longest diameter of the tumor, v (mm), and the length of the shortest diameter of the tumor, w (mm), were measured each time at a fixed time using an electronic vernier caliper, and the tumor volume was calculated using the formula V = ½vw^2^. 

### 4.4. PTX and 3-MA Administration in TNBC Mouse Model

To verify the synergistic effects of 3-MA on PTX, we established another batch of tumor models by tumor mass transplantation. The mice were randomly divided into control group, PTX group (10 mg/kg), and PTX (ca. No. T7191, Sigma-Aldrich, St. Louis, MO, USA, 10 mg/kg) + 3-MA (ca. No. M9281, Sigma-Aldrich, MO, USA, 10 mg/kg) group. The mice in the control group were only given solvent treatment. The mice were sacrificed after 28 days of administration. All the nude mice were anesthetized with 3% sodium pentobarbital, and subcutaneous tumors and para-cancerous and visceral tissues were harvested and stored in a −80 °C freezer.

### 4.5. Histopathological Analysis

The tumor and organ tissues were fixed in 4% paraformaldehyde for 72 h, processed by an automatic tissue processor (Yd-12p, Jinhua Yidi, medical appliance Co., Ltd., Jinhua, China), and embedded in a paraffin block (Yd-6D, Jinhua Yidi, medical appliance Co., Ltd., Jinhua, China). The paraffin blocks were cut into 2-μm-thick sections using a Microm HM 325 microtome (Thermo Scientific, Waltham, MA, USA) and allowed to dry on glass slides overnight at 37 °C. Thereafter, the tissue sections were deparaffinized in xylene and rehydrated through graded ethanol dilutions. Sections of tumor tissues and adjacent tissues were stained with hematoxylin–eosin (H&E) (cat. No. G1120; Solarbio, Beijing, China) according to the manufacturer’s instruction. The slides were observed under the microscope (BX53, Olympus, Tokyo, Japan).

### 4.6. Transmission Electron Microscopy Analysis

To further observe autophagosomes in tumor cells, the tumor tissues were pre-fixed with 2.5% glutaraldehyde. After rinsing to remove residual blood stains, the tissues were trimmed to 1 mm^3^ and placed in fresh fixative for overnight fixation at 4 °C. The tissues were then fixed with 1% OsO4 for 2 h at 4 °C, dehydrated with gradient ethanol, and subsequently embedded in Epon-812 resin. Continuous sections approximately 60 nm thick were prepared using a Leica UC-7 ultramicrotome. The ultrathin sections were loaded onto 100-mesh copper grids and double-stained with 2% uranyl acetate and lead citrate. Finally, observations and imaging were conducted using a transmission electron microscope (JEM-1400plus, JEOL, Tokyo, Japan) at 120 kV.

### 4.7. Immunofluorescence

The tumor tissue sections were dewaxed with xylene and then subjected to gradient alcohol immersion. Subsequently, the expression levels of Ki-67 (Abclonal, Beijing, China), LC3B (Sigma-Aldrich, MO, USA), VPS34 (Cell signaling Technology, Massachusetts, USA), and Caspase-3 (Abcam, Cambridge, UK) in the tumor tissues were detected by immunofluorescence staining. The detailed immunofluorescence staining procedure was performed according to the method described in a previous study [34]. Images were observed and captured using an upright fluorescence microscope (Olympus BX53, Tokyo, Japan).

### 4.8. q-PCR

The total RNA was extracted from the tumor tissue for quantitative polymerase chain reaction (q-PCR) analysis. The total RNA was extracted via the Trizol method. cDNA was synthesized using a reverse transcription kit (ca. No. RR047A, TaKaRa Biotech, Caozin City, Japan) based on 1 μg RNA template. The q-PCR reaction system was established in a 20 μL reaction mixture, which included 10 μL 2 × TB Green (ca. No. RR820A, TaKaRa Biotech, Caozin City, Japan), 1 μL cDNA, 1 μL forward primer, 1 μL reverse primer, and 7 μL ddH_2_O. The relative gene expression levels were calculated with the following formula: X = 2^−∆∆Ct^. The primer sequences are shown in Table 1.

### 4.9. Statistical Analysis

Statistical analysis of the experimental data was performed using SPSS 17.0 statistical software. The mRNA expression level, mouse weight, and tumor volume were detected using One-way ANOVA and Tukey’s post hoc test. The tumor volume measurement results were expressed as Mean ± SEM, and the body weight and q-PCR results were expressed as Mean ± SD. The level of significance was set at * *p* < 0.05, ** *p* < 0.01.

## 5. Conclusions

This study investigated the synergistic antitumor effects of combining the autophagy inhibitor 3-MA with PTX using the TNBC mouse model. Our results demonstrated that PTX monotherapy could inhibit tumor growth, but it activated autophagy in the tumor tissues, potentially inducing drug resistance. In contrast, the combination of 3-MA and PTX effectively reversed PTX-induced autophagy activation and significantly reduced the tumor volume growth rate, exhibiting superior antitumor efficacy compared to the monotherapy and control groups. Mechanistically, the combination therapy synergistically regulated autophagy, apoptosis, and oncogene transcription pathways by inhibiting cell proliferation, inducing apoptosis, and upregulating the expression of c-myc binding protein (*MYCBP)* and autophagy-related genes (*ATG10, DRAM2,* etc.). Furthermore, the successfully established TNBC model in this study provides an experimental platform for subsequent research, while the findings offer a theoretical basis for clinically optimizing PTX treatment regimens, overcoming drug resistance, and improving the prognosis of TNBC patients.

## Figures and Tables

**Table 1:** PCR primer sequences.

Gene Name	Forward	Reverse
*ATG10*	TTCTGAAGTGACGAGACCTGC	AGCCTCGGCTTATAGCACTCA
*FAS*	TATCAAGGAGGCCCATTTTGC	TGTTTCCACTTCTAAACCATGCT
*DRAM2*	GCTGTCCTTGCCTTTAGTATGG	AGATAACCAACAGTAGTCGGACC
*GSTK1*	GGTCCTATGCAGATACCAACAC	GTACTGGCCTTTTCGGGGAA
*MYCBP*	GCTGGACACGCTGACGAAA	TCTAGGCGAAGCAGCTCTATTT
*STK4*	AGCCCTCACGTAGTCAAGTAT	TCTTGTTCCGTAGCCGAATGATA
*STK38*	GCAACCTTATCGCTCAACATGA	CGCGGATCTTCTGAGTCGT
*VPS29*	GCACCAAGGAGAGCTACGAC	TCAGACCGATCTTGAACTGGC
*VPS50*	TATTGTGGAAGGATACGCCAATG	GCTTCGTTGAGTATTCCCTGTG
*CASP1*	CTTGGAGACATCCTGTCAGGG	AGTCACAAGACCAGGCATATTCT
*CASP2*	TACTCCCACCGTTGAGCTGT	CCGTAGCATCTGTGGATAGGC
*CASP8*	CAACTTCCTAGACTGCAACCG	TCCAACTCGCTCACTTCTTCT
*GAPDH*	TGACCTCAACTACATGGTCTACA	CTTCCCATTCTCGGCCTTG

## Data Availability

The original contributions presented in the study are included in the article; further inquiries can be directed to the corresponding authors.
